# Obesity prevention in child care: A review of U.S. state regulations

**DOI:** 10.1186/1471-2458-8-188

**Published:** 2008-05-30

**Authors:** Sara E Benjamin, Angie Cradock, Elizabeth M Walker, Meghan Slining, Matthew W Gillman

**Affiliations:** 1 Obesity Prevention Program, Department of Ambulatory Care and Prevention, Harvard Medical School and Harvard Pilgrim Health Care, 133 Brookline Avenue, 6th Floor, Boston, MA 02215, USA; 2 Harvard Prevention Research Center, Harvard School of Public Health, 677 Huntington Avenue, 7th Floor, Boston, MA 02115, USA; 3 Center for Children's Health Innovation, Nemours Health and Prevention Services, 252 Chapman Rd., Suite 200, Newark, DE 19709, USA; 4 Department of Nutrition, 260 Rosenau Hall, University of North Carolina at Chapel Hill, Chapel Hill, NC 27599, USA

## Abstract

**Objective:**

To describe and contrast individual state nutrition and physical activity regulations related to childhood obesity for child care centers and family child care homes in the United States.

**Methods:**

We conducted a review of regulations for child care facilities for all 50 states and the District of Columbia. We examined state regulations and recorded key nutrition and physical activity items that may contribute to childhood obesity. Items included in this review were: 1) Water is freely available; 2) Sugar-sweetened beverages are limited; 3) Foods of low nutritional value are limited; 4) Children are not forced to eat; 5) Food is not used as a reward; 6) Support is provided for breastfeeding and provision of breast milk; 7) Screen time is limited; and 8) Physical activity is required daily.

**Results:**

Considerable variation exists among state nutrition and physical activity regulations related to obesity. Tennessee had six of the eight regulations for child care centers, and Delaware, Georgia, Indiana, and Nevada had five of the eight regulations. Conversely, the District of Columbia, Idaho, Nebraska and Washington had none of the eight regulations. For family child care homes, Georgia and Nevada had five of the eight regulations; Arizona, Mississippi, North Carolina, Oregon, Tennessee, Texas, Vermont, and West Virginia had four of the eight regulations. California, the District of Columbia, Idaho, Iowa, Kansas, and Nebraska did not have any of the regulations related to obesity for family child care homes.

**Conclusion:**

Many states lack specific nutrition and physical activity regulations related to childhood obesity for child care facilities. If widely implemented, enhancing state regulations could help address the obesity epidemic in young children in the United States.

## Background

Rates of obesity in children continue to rise in the United States and abroad [1-4]. Even among preschool-aged children the prevalence of obesity is alarmingly high, with 26.2% of children aged 2 through 5 years in the United States classified as either overweight or obese [2]. Even in childhood, obesity is associated with a variety of adverse health consequences that can include Type II diabetes mellitus [5,6], hypertension and hyperlipidemia [6,7], asthma and sleep apnea [8], early maturation [9], lower self-esteem,[10] and psychosocial stress [11,12]. Additional research has identified the preschool period as a critical time for growth, development, and risk of later obesity [13-15].

While there are genetic factors related to childhood obesity, diet and physical activity-related causes are modifiable and have therefore been targets of obesity prevention efforts and research. Associations between dietary intake and obesity have been examined in numerous studies [16-21]. Intake of sugar sweetened beverages and high fructose corn syrup [22,23] may be contributors to the obesity epidemic, as the increases in consumption show a pattern consistent with the rise in obesity [16-19]. Other studies corroborate this finding and report that sweetened beverage consumption, including soft drinks and fruit juice, has increased in all children, including toddlers [23,24], and is related to childhood obesity [25]. Studies examining the relationship between fruit juice intake and childhood obesity have shown mixed results [26-28].

Adult behaviors may interfere with a child's ability to respect hunger and satiety cues. There is some evidence that restrictive feeding and forcing children to eat are related to childhood obesity [29-34]. Moreover, using food as a reward may also have a negative effect on children's weight status [35,36]. Additionally, there is strong evidence that breastfeeding has a protective effect against later childhood and adolescent obesity [37-40].

Strong evidence also links childhood obesity to television viewing both through observational studies and randomized controlled trials [41-47]. Even among preschool-aged children television viewing is associated with risk of obesity [41,44-47]. Little is known about the contribution of computer use and its relationship to obesity in young children; one recent study found an association between computer use and adiposity in preschool-aged children [47]. In addition to television and other screen time, researchers have associated physical activity with obesity in young children, with low levels of physical activity observed among preschool-aged children [48], and in particular, preschool-aged girls [49]. Burdette and Whitaker found that play, which can involve any type of physical movement, is on the decline among children of all ages and may contribute to increases in sedentary activity and obesity [50].

Children under the age of five years have recently been a focus of obesity prevention efforts [51,52]. In the United States and in many other countries, a large percentage of young children are in some form of child care, and the amount of time children spend in child care each week has increased in recent years [53-57]. In the United States, the 2001 National Household Education Survey found that 74% of children ages three to six are in some form of non-parental care and 56% are in center-based child care [58]. Similarly, approximately two thirds of infants are cared for by someone other than a parent, while half attend center-based care [59]. Child care facilities may serve as home-away-from-home settings, where children adopt early nutrition, physical activity, and television viewing behaviors. These behaviors are often a result of interactions with parents and other caregivers [60]. Young children in particular are more likely to be influenced by adults in an eating environment [61]. Moreover, preschool-aged children may consume 50% to 100% of their Recommended Dietary Allowances in child care settings [62], placing a great deal of responsibility on the child care facility to provide nutritionally adequate, healthful food.

Child care facilities are in a unique position to support and facilitate healthful eating and promote physical activity for young children, and a small number of studies have targeted child care settings for nutrition promotion [63-67] and obesity prevention [68-72]. While individual and environmental intervention efforts represent one approach to improving health outcomes of children in child care, broad-sweeping state policy changes may play a complementary or perhaps primary role in childhood obesity prevention.

In the United States, regulation of child care facilities is the responsibility of the individual state and the District of Columbia, and each has an agency responsible for oversight and enforcement of these regulations [73]. As a result, regulations for child care facilities vary considerably by state. A recent report of state regulations by the National Association of Child Care Resource & Referral Agencies (NACCRRA) scored states on a variety of health and safety benchmarks including: staff to child ratios, Sudden Infant Death Syndrome (SIDS) prevention, hand washing and diapering, playground surfaces, and emergency preparedness [74]. Nutrition and physical activity, however, were not included in this report. We conducted a review of state nutrition and physical activity regulations related to childhood obesity for child care facilities in the United States and present results of a state-by-state comparison. Although one publication reported tallies of a small number of state regulations related to nutrition and physical activity [75], no comprehensive review has examined obesity-related regulations for child care facilities.

## Methods

### Subjects

We collected data on individual state regulations for child care facilities between January and August of 2007 from the National Resource Center for Health and Safety in Child Care website available at . The National Resource Center for Health and Safety in Child Care maintains a public access database of licensing regulations for all fifty US states and the District of Columbia. Regulations are updated when changes are made and reflect the most current regulations available from states.

### Selection of key items

We reviewed state regulations for reference to seven key nutrition and physical activity items related to childhood obesity. The items have a documented relationship to childhood obesity in the research literature, and are likely contributors to diet quality and activity level. Items included in this review were: 1) Water is freely available [76,77]; 2) Sugar sweetened beverages are limited [18,19,28,78-82]; 3) Foods of low nutritional value are limited [81,83,84]; 4) Children are not forced to eat [29-34]; 5) Food is not used as a reward [35,36]; 6) Support is provided for breastfeeding and provision of breast milk [37-40]; and 7) Screen time is limited [41-45]; and 8) Physical activity is required daily (minutes per day) [48-50].

During the review of state regulations, we found more than 50 nutrition and physical activity regulations. The majority of food-related regulations were more associated with food safety and sanitation and the majority of physical activity-related regulations were related to injury prevention. Of those regulations not related to food safety, sanitation, or injury prevention, we selected regulations more closely associated with obesity. A number of regulations were not included in this review (e.g., mandating provision of fruits and vegetables, prohibiting vending machines) because either the regulation was only mentioned in 1 or 2 states or the research literature was inconclusive on the relationship between the item and childhood obesity.

### Review of regulations

We reviewed regulations for child care facilities for all 50 US states and the District of Columbia between June and August of 2007. Most states license 2 classes of child care facilities: child care centers and family child care homes. Child care centers by definition care for greater numbers of children and typically have more employees than family child care homes. A second key difference is that family child care homes are located in the residence of the owner and operator of the child care facility, who is often the only provider of care. States have varying definitions for the maximum number of children allowed to receive care in a family child care home, but typically limit enrollment to six or fewer children. In certain cases, states refer to these classifications differently. Nine states have only 1 regulation that governs both child care centers and family child care homes, 16 states have a separate regulation for centers and one for family child care homes, and 26 states have regulations for more than 2 distinct classes of facilities. We grouped additional facility types into one of the 2 main classes (child care centers or family child care homes). For example, Hawaii issues a license for 4 types of facilities, which we grouped into 2 types as follows: "group child care centers" and "infant and toddler child care centers" were classified as child care centers, and "family child care homes" and "group child care homes" were classified as family child care homes.

## Results

States varied considerably in their nutrition and physical activity regulations related to obesity for both child care centers (Table 1), and family child care homes (Table 2).

**Table 1:** US state nutrition and physical activity regulations related to obesity for child care centers, 2007

State	Year of last update	Water freely available	Sugar sweetened beverages limited	Foods of low nutritional value limited	Children not forced to eat	Food not used as reward	Support provided for breastfeeding and provision of breast milk	Screen time limited	Physical activity required daily- minutes per day specified
AL	2001	X			X	X		X*	
AK	2006				X			X	X
AZ	2004	X			X			X†	
AR	2006	X							
CA	2005	X	X‡	X‡					
CO	2005/7	X						X§	
CT	2005	X							
DC	1987								
DE	2005	X			X		X	X§	X
FL	2005	X							
GA	1998	X	X||	X||	X			X*	
HI	2002	X			X	X			
ID	2006								
IL	2005	X			X	X		X*	
IN	2003	X	X¶			X	X	X*	
IA	2006	X				X			
KS	1990	X			X				
KY	2001	X							
LA	2003	X							
ME	2006	X			X				
MD	2006	X			X				
MA	1997	X			X				
MI	2006						X	X*	
MN	1994	X							
MS	2006				X	X	X	X	
MO	2002	X		X**	X				
MT	2006	X							
NE	1998								
NV	2004	X	X||	X||	X	X			
NH	2006				X				
NJ	2005	X			X				
NM	2006	X	X					X	
NY	2005	X			X		X		
NC	2006	X	X||	X||	X				
ND	1999	X			X				
OH	2007	X					X		
OK	2006	X			X				
OR	2003	X	X††	X||	X				
PA	2005	X			X				
RI	1993	X							
SC	2005	X			X			X*	
SD	2004				X				
TN	2006	X		X	X	X‡‡	X	X*	
TX	2006	X			X	X		X	
UT	2006	X			X				
VT	2001	X			X			X*	
VA	2005	X			X		X		
WA	2006								
WV	2007	X			X		X	X*	
WI	2005	X			X			X*	
WY	2001				X	X			

* Alternate activity must be provided (GA, IL, IN, MI, TN, VT, WA, WV, WI) or children are not required to watch (AL, IN, SC, WI)

† Television cannot be on when a child is sleeping

‡ No corn syrup served to infants

§No television without parental permission

|| Served on special occasions only (GA, NC) or occasionally (OR) and in addition to required meals and snacks (GA, NV, OR)

¶No ades, drinks, soft drinks, or powders served or accessible to children

** Snacks of fruit juice, raw fruits and vegetables, milk, crackers, cheese, peanut butter, or similar nutritious foods shall be served

†† For infants less than 12 months of age

‡‡ Desserts and sweets not used as a reward

**Table 2:** US state nutrition and physical activity regulations related to obesity for family child care homes, 2007

State	Year of last update	Water freely available	Sugar sweetened beverages limited	Foods of low nutritional value limited	Children not forced to eat	Food not used as reward	Support provided for breastfeeding and provision of breast milk	Screen time limited	Physical activity required daily- minutes per day specified
AL	2001				X				
AK	2006				X			X	X
AZ	2004	X	X*	X	X				
AR	2006	X			X				
CA	2006/7								
CO	2005	X			X			X†	
CT	2005	X							
DC	1987								
DE	2002							X	X
FL	2004	X							
GA	1991/4	X	X‡	X‡	X			X§	
HI	2002	X			X	X			
ID	2006								
IL	2003	X			X				
IN	2001	X							
IA	2004								
KS	1990								
KY	2001/3	X							
LA	2000	X							
ME	2006	X			X				
MD	2006				X				
MA	2003	X			X				X
MI	2006	X			X				
MN	1985	X							
MS	2006				X	X	X	X	
MO	2002	X		X||	X				
MT	2006	X						X	
NE	1999								
NV	2004	X	X‡	X‡	X	X			
NH	2006				X				
NJ	2004	X			X				
NM	2006		X						
NY	2005	X			X		X		
NC	2006	X	X‡	X‡	X				
ND	1999				X				
OH	2007	X					X		
OK	2006	X			X				
OR	2006	X	X¶	X‡				X	
PA	2005	X			X				
RI	1988/90	X						X	
SC	2005	X			X			X§	
SD	2004				X				
TN	2005		X**	X**	X			X§	
TX	2006	X			X	X		X	
UT	2002	X			X				
VT	2001	X		X††	X			X§	
VA	1993	X			X				
WA	2006	X						X§	
WV	2007	X		X‡‡	X			X	
WI	2005	X			X			X§	
WY	2001				X	X			

* Not served in place of juice

† No television without parental permission

‡ Served on special occasions only (GA, NC) and in addition to required meals and snacks (GA, NV)

§Alternate activity must be provided (GA, TN, VT, WA, WI) or children are not required to watch (SC, WI)

|| Snacks of fruit juice, raw fruits and vegetables, milk, crackers, cheese, peanut butter, or similar nutritious foods shall be served

¶For infants less than 12 months of age

** Carbonated drinks, fruit-flavored drinks, imitation milk drinks, and candy shall not be served as snack foods

†† Emphasis placed on foods unprocessed, and low in salt and sugar

‡‡ Limit snack foods high in salt and sugar

### Water is freely available to children

Ensuring availability of water was the most common regulation. Forty-one states (80%) had regulations to ensure that water was available to all children in child care centers. Thirty-four (67%) required availability of water in family child care homes. In addition, the wording of this regulation tended to be very similar from state to state. Generally, states required water to be "freely available to all children at all times". In some cases, states required child care staff to offer water to children between meals and snacks or at frequent intervals (centers: GA, IN, LA, MN, MT, NV, NY, NC, OK, VA, WI; homes: LA, MN, NV, NY, NC, OK, VA) or during outdoor play or in warm weather (centers: PA, UT, VA, VT; homes: LA, PA, UT, VT). A few states either encouraged (centers: OR, WI; homes: WI, WV) or discouraged (centers: LA, WV) giving water to infants in child care.

### Sugar sweetened beverages are limited

Seven states (14%) restricted sugar sweetened beverages in both child care centers and family child care homes. A number of states specified that sugar sweetened beverages could not take the place of healthier beverages (centers: GA, NV, NM; homes: AZ, GA, NV, NM, TN) or could be served only on special occasions (centers: GA, NC; homes: GA, NC).

### Foods of low nutritional value are limited

Although states define these foods differently, nine states (18%) limited foods of low nutritional value in centers while seven (14%) restricted those foods in family child care homes. North Carolina provided examples of foods with low nutritional value, listing gelled desserts, popcorn, desserts, and potato chips as foods that centers could serve only occasionally and not in place of nutritious foods. Tennessee restricted "highly inappropriate foods" or those foods high in sugar and high in fat. For family child care homes, but not centers, Arizona limited high fat, high sugar foods to no more than two times per week. West Virginia regulations stated that centers must limit snack foods with high sugar and salt. Two states provided examples of healthful foods that should be served in child care: Vermont encouraged family child care homes to emphasize unprocessed, low sugar, and low salt foods, while Missouri required child care centers to serve snacks of fruit juice, raw fruits and vegetables, milk, crackers, cheese, peanut butter, or "similar nutritious foods."

### Children are not forced to eat

The second most common regulation prohibited forcing children to eat. We found this regulation most often under the heading of discipline, rather than nutrition or food service within state regulations. Thirty-two states (63%) did not allow providers to force children to eat in both child care centers and family child care homes.

### Food is not used as a reward

Ten states (20%) did not allow food to be used as a reward in child care centers. Tennessee specified that desserts and other sweets should not be used as a reward in child care centers. Five states (10%) did not allow providers to use food as a reward in family child care homes. Since Washington state prohibited using food as a reward only when toilet training children, it was not included as having a regulation prohibiting using food as a reward.

### Support is provided for breastfeeding and provision of breast milk

We found regulations expressing support for breastfeeding and provision of breast milk in only 9 states (18%) for child care centers. Indiana, however, also required parents to have a written breastfeeding agreement on file, to provide breast milk in single serving sterilized bottles or sterile nurser bags labeled with the child's name, the date and the time collected, and to keep milk at 41 degrees or less during storage and transport. Three states (6%) included a statement of support for breastfeeding in their regulations for family child care homes.

### Screen time is limited

Seventeen states (33%) regulated screen time in child care centers and 15 (29%) in family child care homes. For child care centers, 7 states limited the number of screen time hours children received in a day. Mississippi, New Mexico, and Delaware limited viewing to 1 hour, Alaska 1.5 hours, and Georgia and Tennessee required less than 2 hours of screen time per day. Texas limited screen time viewing to less than 5 hours per week. In addition, screen time was not allowed for children less than 2 years of age in Delaware or for infants in Mississippi child care centers. For family child care homes, 6 states set limits on daily screen time. Mississippi limited screen time viewing to 1 hour, Alaska 1.5 hours, and Delaware, Georgia, and Oregon limited screen time to 2 hours per day. Texas limited viewing to less than 5 hours per week. Screen time was not allowed for infants in family child care homes in Mississippi. Additionally, Delaware recently proposed to decrease screen time to 1 hour per day in family child care homes.

A number of states required child care facilities to offer children an alternative to television viewing (centers: GA, IL, IN, MI, TN, VT, WA, WV, WI; homes: GA, TN, VT, WA, WI), or specified that children were not required to watch television (centers: AL, IN, SC, WI; homes: SC, WI). Delaware and Colorado are the only 2 states to require parental permission to use television during child care hours.

### Physical activity is required daily (minutes per day specified)

Only 3 states required a specified number of minutes of physical activity per day. Alaska and Delaware mandated that children engage in 20 minutes of moderate to vigorous physical activity for every 3 hours the child care centers and family child care homes were open between the hours of 7:00 am and 7:00 pm. Currently, Massachusetts requires 30 minutes of daily physical activity for all children in family child care homes. However, Massachusetts recently proposed an increase to 60 minutes of physical activity daily that would be required for children in both child care centers and family child care homes. If this proposal is approved, the new regulation will take effect in late 2008.

## Discussion

In this review of state regulations for child care facilities in the United States, we found that most states had few nutrition and physical activity regulations related to obesity for child care centers and family child care homes. Tennessee had 6 of the 8 obesity regulations for child care centers, and Delaware, Georgia, Indiana, and Nevada had 5 of the 8 regulations. On the other hand, the District of Columbia, Idaho, Nebraska and Washington had none of the 8. For family child care homes, Georgia and Nevada had 5 of the 8 regulations; Arizona, Mississippi, North Carolina, Oregon, Tennessee, Texas, Vermont, and West Virginia had 4 of the 8 regulations. California, the District of Columbia, Idaho, Iowa, Kansas, and Nebraska did not have any of the regulations for family child care homes.

In addition to the overall dearth of nutrition and physical activity regulations related to obesity, the regulations that do exist may be difficult to interpret by child care providers. The regulation related to water, for example, generally did not specify where and how water should be provided to children, only that it should be freely available. Other regulations may be difficult to decipher as well. A number of states prohibited forcing children to eat. This regulation, however, was mostly associated with discipline, and may not be applied to adult-child interactions during meals and snacks (e.g., requiring children to "clean their plates" or take a "no thank you" bite). This regulation may have a greater impact if states explicitly stated that this regulation applied to all eating occasions. The screen time and the physical activity regulations, however, may be easier to interpret and implement because they offer more precise guidelines.

We also examined regulations related to vending machines in child care facilities. We did not report results on vending machines because there is limited data linking vending machines to obesity in children. We did, however, find that only 4 states (8%) restricted or prohibited vending machines at the child care center. Two of these states, Mississippi and Georgia, also regulated vending machines in family child care homes. Alabama and Georgia prohibited vending machines in any areas used by children, while Louisiana required them to be outside of children's play areas. Mississippi required food in the vending machine to meet nutritional guidelines. We present this information on vending machines as a supplement to our review of nutrition and physical activity regulations related to obesity for child care facilities.

Given the overall absence of regulations or lack of clear and specific nutrition and physical activity regulations related to childhood obesity, a number of states could enhance their regulations for child care facilities. Reviewing and revising regulations on a regular basis helps ensure that regulations reflect current best practices related to childhood obesity. As additional evidence becomes available, states should revise regulations for both child care centers and family child care homes to reflect this new information. In this review, we found that 8 states had regulations dating back to the 1980s or 1990s. In a recent publication, Story et al. [75] contend that inadequate nutrition and physical activity regulations represent a missed opportunity for childhood obesity prevention. Moreover, they argue that simple, easy to follow nutrition and physical activity regulations, such as prohibiting sugar-sweetened beverages and requiring physical activity daily may help promote healthy weight in young children in child care [75]. If widely implemented, enhancing state regulations could be one way of improving weight-related nutrition and physical activity behaviors in preschool-aged children in the United States.

Generally, regulations for family child care homes tended to be less stringent than centers. In a few instances, however, regulations for family child care homes were more robust than child care centers. For example, Washington required homes but not centers to offer water at frequent intervals to children, and Arizona limited sugar sweetened beverages and foods of low nutritional value in family child care homes, but not in child care centers. Additionally, Oregon limited screen time in family child care homes but not centers. Although family child care homes are generally considered a more informal child care setting, states should provide the same regulations for both family child care homes and centers. This helps to ensure consistent quality of care, and minimizes variation among child care providers within a given state.

A limitation of this review is the ever-evolving nature of state regulations. States may have revised regulations for child care centers or family child care homes since our last review, or may be in the process of updating their regulations. Given the current national call-to-action to address childhood obesity in both schools and child care settings, states may be more likely than ever before to enact nutrition and physical activity regulations to help prevent obesity. In addition, cities or other geographic areas within a state have the power to regulate child care facilities in their jurisdiction. New York City, for example, recently enacted nutrition and physical activity regulations in Article 47 of the New York City health code that were more stringent than those for New York State. New York City is leading the way for other cities who may want to enact new regulations for child care facilities that go beyond their state regulations.

## Conclusion

In this review of US state regulations for child care we found that most states had few regulations related to obesity for child care centers and family child care homes. This is the first comprehensive review of nutrition and physical activity regulations related to childhood obesity for child care facilities, and further exploration is needed. Recently, researchers have attempted to classify and categorize state level policy related to physical education and nutrition services in school settings [85,86], but the researchers did not extend the scope of their work to include state regulations governing child-care facilities. A logical next step would be to conduct a similar study classifying obesity regulations for child care facilities in the United States. In addition, existing child care regulations could be compared to federal recommendations to assess how states regulations compare to national standards.

The mere existence of a regulation, however, does not necessarily ensure compliance or enforcement. Frequency of compliance checks vary from state to state. For child care centers, 29 states visit centers one time per year, 16 states visit 2 times per year, and 6 states visit 3 or more times per year for routine inspections. For family child care homes, states assess compliance less frequently (range: every 6 months to 10 years). Some states do not require routine inspections of family child care homes unless a formal complaint is filed. Future studies should explore not only the presence of a regulation but also implementation, compliance, enforcement, and unintended consequences of the regulation, as well as the overall effect on child health outcomes. This review and report of state nutrition and physical activity regulations for child care facilities in the United States represents the first step in policy-level approaches to address childhood obesity. Results of this review, coupled with a growing interest in obesity prevention in child care settings, may inspire states to improve and enhance their nutrition and physical activity regulations related to obesity and could spark additional research in this area to help evaluate the impact of these regulations.

## Competing interests

The authors declare that they have no competing interests.

## Authors' contributions

SEB conceived of the idea for this manuscript, reviewed state regulations, and drafted the article. AG provided scientific input in the development of this project and provided feedback on multiple drafts of the article. EW contributed to the review of state regulations and provided feedback on multiple drafts of the article. MS contributed to the review of state regulations and provided feedback on multiple drafts of the article. MWG provided scientific oversight of this work and provided extensive feedback throughout each state of development of the manuscript. All authors read and approved the final manuscript.

## Pre-publication history

The pre-publication history for this paper can be accessed here:


